# Handover Optimization Algorithm Based on T2RFS-FNN

**DOI:** 10.1155/2022/6293192

**Published:** 2022-12-14

**Authors:** Yong Chen, Kaiyu Niu, Wei Zhang

**Affiliations:** ^1^School of Electronic and Information Engineering, Lanzhou Jiaotong University, Lanzhou 730070, China; ^2^School of Traffic and Transportation, Lanzhou Jiaotong University, Lanzhou 730070, China

## Abstract

As a key technology for highly reliable communication in the fifth generation mobile communication for railway (5G-R) high-speed railway wireless communication system, once the handover fails, it will pose a serious risk to the safe operation of high-speed railway. As the speed of high-speed trains continues to increase, the handover will become more frequent, and how to improve the success rate of the handover is a key problem that needs to be solved. In this paper, we proposed an optimization algorithm based on the interval type 2 feature selection recurrent fuzzy neural network (T2RFS-FNN), which is a recurrent fuzzy neural network with interval type 2 feature selection, to address the problem of fixed hysteresis threshold and single consideration for the handover algorithm between the control plane and the user plane of the high-speed railway under 5G-R. The algorithm integrates reference signal receiving power (RSRP). Reference signal receiving quality (RSRQ) and throughput to optimise the hysteresis threshold. First, a feedforward neural network structure is designed to implement fuzzy logic inference, and an interval type-two Gaussian subordination function is used to improve the nonlinear expressiveness of the model. Then, a feature selection layer is added to determine the output of the affiliation function, which completes the optimization of the hysteresis threshold and overcomes the drawback of the fixed hysteresis threshold of the handover algorithm. Finally, simulation analysis of the control-plane and user-plane handover algorithms is carried out separately. The results show that the proposed method can effectively improve the success rate and reduce the ping-pong handover rate compared to the comparison algorithms. The results provide a theoretical reference for the speedup of high-speed railway trains and the evolution of the global system for mobile communications for railway (GSM-R) to 5G-R.

## 1. Introduction

At present, China's high-speed railway wireless communication system uses GSM-R wireless system, but GSM-R belongs to a 2G narrowband system, and this system can no longer meet the requirements of high-speed railway wireless communication for low latency and high reliability [1]. High-speed railway wireless communication system will evolve to 5G-R. The 5 G high-speed railway mobile communication system has the advantages of a high information-data transmission rate, low transmission delay, and high system capacity [2]. During train operation, in order to maintain uninterrupted communication, the train needs to constantly handover the area to the base station. As a key technology for 5G-R communication, handover is crucial to ensure traffic safety [3], and as high-speed trains continue to increase speed, handover will become more frequent, so how to improve the success rate of handover is a key problem that needs to be solved.

The literature [4] proposes a fuzzy neural network (FNN)-based handover algorithm that uses fuzzy neural networks in the handover decision to optimise the handover performance, but the algorithm suffers from co-channel interference and the network's poor handling of nonlinear data. In [5], a handover scheme based on CoMP and bicast technology in C/U separation architecture is proposed to establish a connection at the target user plane before disconnecting the source base station by using cooperative multipoint transmission and bicast technology, but the scheme suffers from the problem of fixed hysteresis threshold. Subsequently, in [6], a handover scheme with dual antennas and beam focusing is proposed, where dual antennas are installed at the front and rear of the vehicle to ensure the communication link in a C/U separated architecture, while beam focusing is used to improve the signal quality, but the scheme is less effective in optimising the handover success rate. The literature [7] proposes handover algorithms for the control plane and user plane, respectively, by using a speed-based handover scheme for the control plane and a CoMP-based handover scheme for the user plane, but the scheme suffers from the problem of a single consideration in the user plane handover algorithm. The literature [8] proposes a Bayesian regression-based handover algorithm that uses a Bayesian regression model to predict the RSRP of the control plane and the user plane to achieve early handover, but the method is less capable of handling nonlinear data.

In summary, this paper proposes an optimization algorithm based on the recurrent fuzzy neural network of interval type 2 feature selection recurrent fuzzy neural network (T2RFS-FNN) for the control-plane and user-plane handover algorithm with fixed hysteresis thresholds and a single consideration factor under 5G-R. The main work carried out in this paper includes the following: (1) constructing a fuzzy neural network model for predicting and optimising the hysteresis threshold, using interval binary affiliation functions, and adding a feature selection layer to improve the network model's ability to optimise the hysteresis threshold. (2) Adjusting the inputs to the network to optimise the threshold between the control plane and the user plane, considering the functional differences between the control plane and the user plane. The performance of the designed handover optimization algorithm is simulated in terms of handover success rate and ping-pong handover rate. The results show that the proposed T2RFS-FNN handover optimization algorithm for the high-speed 5G-R railway can effectively improve the handover success rate and reduce the ping-pong handover rate compared with the traditional A3 handover algorithm.

The remainder of this paper is organized as follows: Section 2 presents the basic theory of handover.  Section 3 introduces the T2RFS-FNN-based optimization algorithm for handover. The handover algorithm performance indicators are provided in Section 4. Experimental results and analysis are presented in Section 5. Finally, the paper is concluded in Section 6.

## 2. Basic Theory of Handover

### 2.1. Handover

**Figure 1:**
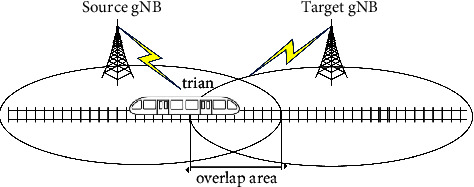
Schematic diagram of high-speed railway handover.

To ensure the continuity of communication, the train must disconnect from the source cell and establish a connection with the target cell when entering the signal coverage overlap area, a process called handover, which is shown in Figure 1 [9].

### 2.2. Traditional A3 Event Crossing Handover Algorithm

**Figure 2:**
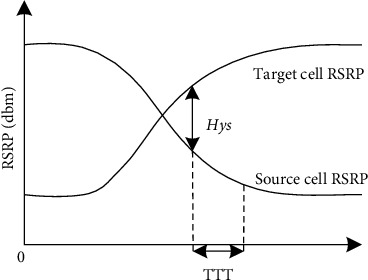
Diagram of A3 algorithm.

The algorithm used for the handover under 5G-R is the A3 event-based crosszone handover algorithm [10]. The core idea is that the reference signal receiving power RSRP or reference signal receiving quality RSRQ of the source and target cells are measured periodically, and if equation (1) is satisfied, when the RSRP of the target cell is higher than the RSRP value of the source cell by Hys, then the UE performs a crosszone handover after triggering the time delay TTT, as shown in Figure 2. The triggering conditions for the A3 event.

(1) $$\begin{array}{c} Mt-Hys>Ms\text{,} \end{array}$$

where Mt and Ms are the signal strengths of the target and source cells, respectively, and Hys is the hysteresis threshold.

### 2.3. Handover in C/U Separated Architectures

**Figure 3:**
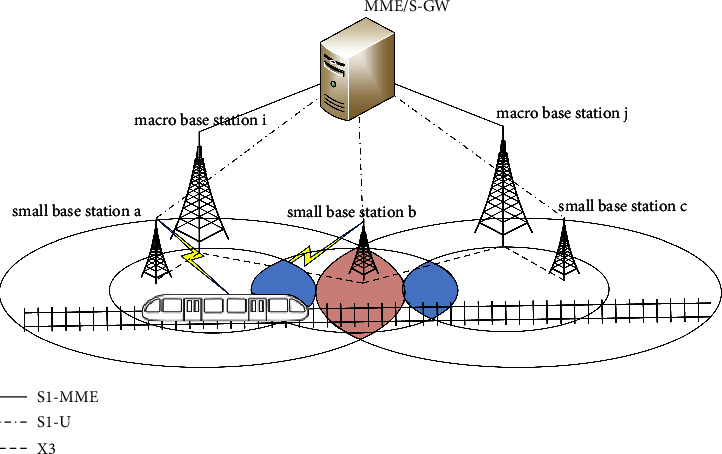
Schematic diagram of handover in the framework of separation of C/U.

The signal coverage overlap zone exists between adjacent base stations, where the blue area is the signal coverage overlap zone of adjacent small base stations and the red area is the signal coverage overlap zone of adjacent macro base stations, and the train needs to disconnect from the current base station, and establish a connection with the target base station when entering the signal coverage overlap zone for handover. The schematic diagram of handover, as shown in Figure 3, where the base station communicates with the MME/S-GW through the *S*1 interface and the macro base station communicates with the small base station through the *X*3 interface [11].

In the 5G-R wireless communication system, the key technology is the network architecture based on C/U separation [12]. The C/U separation architecture in the 5G-R network is divided into macrobase station handover and small base station handover [7], where both the macrobase station handover and small base station handover algorithms use the A3 event-based handover algorithm.

## 3. T2RFS-FNN-Based Optimization Algorithm for Handover

To address the problems of fixed hysteresis thresholds and single considerations in control-plane and user-plane handover algorithms, this paper proposes a crosszone handover optimization algorithm based on interval type 2 feature selection recurrent fuzzy neural network (T2RFS-FNN). The algorithm uses a feedforward neural network structure to implement FL inference, adopts an interval type II Gaussian subordination function to improve the nonlinear expression capability of the model, adds a feature selection layer to determine the output of the subordination function, and uses uncertainty inference to perform fuzzy inference to complete the optimization of the hysteresis threshold. The control plane and user plane are separated in the 5G-R overzone handover process, with the macrobase station and MME forming the control plane for reliable transmission of critical control signalling, and the user plane with high throughput requirements being carried by the 5G base station. Therefore, this paper adopts the T2RFS-FNN-based crossover handover optimization algorithm to optimize the handover hysteresis threshold by considering RSRP, RSRQ, and throughput, and adjusts the inputs of the network to optimize the threshold values of the control plane and user plane, respectively, due to the functional differences between the control plane and the user plane. This means that the hysteresis threshold is optimised by considering RSRP, RSRQ, and throughput for control-plane handover and by considering RSRP, RSRQ, and throughput for user-plane handover.

### 3.1. Network Structure

**Figure 4:**
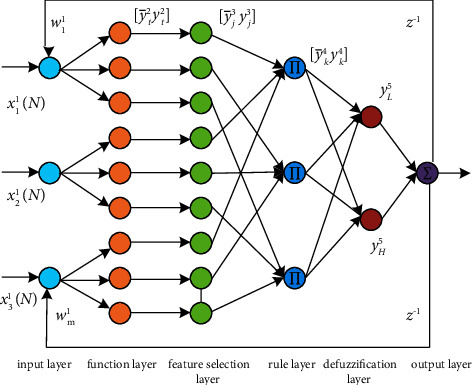
T2RFS-FNN network structure.

The network structure of the method in this paper is shown in Figure 4. The network is structured as a cyclic. The structure of the loop neural network implements the inference process of interval type II fuzzy logic, which consists of six layers of neuron nodes, namely the input layer, the subordinate degree function layer, the feature selection layer, the rule layer, the defuzzification layer, and the output layer [13].

The first layer is the input layer, which contains three input parameters, namely RSRP, RSRQ, and throughput received by the train from the target base station, and the input-output relationship for each node in the input layer is as follows:

(2) $$\begin{array}{c} {node}_{i}^{1}\left(N\right)=x_{i}^{1}\left(N\right)w_{i}^{1}\left(N\right)y_{o}^{6}\left(N-1\right)\text{,}\\ y_{i}^{1}\left(N\right)=f_{i}^{1}\left({node}_{i}^{1}\left(N\right)\right)={node}_{i}^{1}\left(N\right)\text{.} \end{array}$$

Equation (2) represents the input of nodes and the output of the first layer network, and node_*i*_^1^ denotes the input of the node, *i* = 1,2,…, *m*, *N* is the sampling period, is the network output of the previous sampling period, *w* denotes the cyclic weight, and *y*_*i*_^1^(*N*) is the output of the input layer.

**Figure 5:**
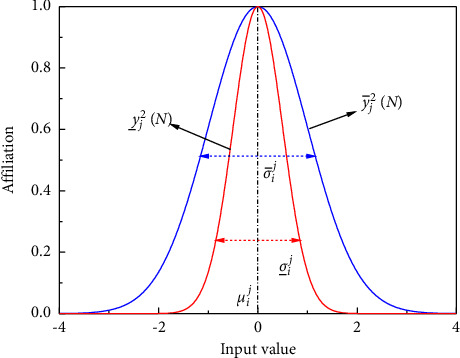
Schematic diagram of type-2 Gaussian membership function.

The second layer is the layer of affiliation function, where each node in this layer performs a Gaussian interval type II affiliation function, which is mapped to a region by the interval type II affiliation function, rather than just to an exact value on the interval [0, 1], where the values in the region are more random, as shown in Figure 5.

For each node *j* in the layer:

(3) $$\begin{array}{c} {\bar{y}}_{j}^{2}\left(N\right)=\exp\left({-\frac{1}{2}\left(\frac{x_{i}^{2}\left(N\right)-u_{i}^{j}}{{\bar{\sigma }}_{i}^{j}}\right)}^{2}\right)\text{,} \end{array}$$

(4) $$\begin{array}{c} {\underline{y}}_{j}^{2}\left(N\right)=\exp\left({-\frac{1}{2}\left(\frac{x_{i}^{2}\left(N\right)-u_{i}^{j}}{{\underline{\sigma }}_{i}^{j}}\right)}^{2}\right)\text{.} \end{array}$$

Equations (3) and (4) represent the uplink and downlink output of the layer 2 node, where *x*_*i*_^2^(*N*)=*y*_*i*_^1^(*N*) is the input to the nodes in this layer, *y*_*i*_^2^(*N*) is the output of the second layer, exp is the exponential function, (*i* = 1,…, *m*, *j* = 1,…, *n*) denoted as the average of the Gaussian functions in the *j-*th term associated with the *i*-th input, the *σ*_*i*_^*j*^ are denoted as the standard deviations of the upper and lower Gaussian affiliation functions in the *j-*th term relative to the *i*-th input, respectively.

The third layer is the feature selection layer, for each node in this layer, and the feature degree is measured by using a feature selection algorithm. Equations (5) and (6) are executed for the *j-*th node:

(5) $$\begin{array}{c} {\bar{y}}_{j}^{3}\left(N\right)=\left\{\begin{array}{cc} {\overset{¯}{a}}_{j}\left(N\right)\leq {\overset{¯}{T}}_{J}\text{,}\\ {\overset{¯}{y}}_{j}^{2}\left(N\right)\left[1-\exp\;\left(-{\overset{¯}{\beta }}_{j}\left(n\right)\right)\right]\text{,} & {\bar{a}}_{j}\left(N\right)>{\bar{T}}_{J}\text{,} \end{array}\right. \end{array}$$

(6) $$\begin{array}{c} {\underline{y}}_{j}^{3}\left(N\right)=\left\{\begin{array}{cc} {\underline{a}}_{j}\left(N\right)\leq {\underline{T}}_{J}\text{,}\\ {\underline{y}}_{j}^{2}\left(N\right)\left[1-\exp\;\left(-{\underline{\beta }}_{j}\left(n\right)\right)\right]\text{,} & {\underline{a}}_{j}\left(N\right)>{\underline{T}}_{J}\text{,} \end{array}\right.\\ {\underline{a}}_{j}\left(N\right)=1-\exp\;\left(-{\underline{\beta }}_{j}\left(N\right)\right)\text{,} \end{array}$$

where *x*_*i*_^3^(*N*) is the input to the layer, *y*_*i*_^3^(*N*) is the output of the nodes in the layer, and *a*_*i*_(*N*) represents the feature degree of each node. *T*_*i*_(*N*) is denoted as [${\bar{T}}_{i}\left(N\right){\;\underline{T}}_{i}\left(N\right)$]*ϵ*[0, 1] and represents the threshold for judging unfavourable nodes. The feature selection algorithm is described as follows: if ${\bar{a}}_{i}\left(N\right)$ is equal to 1, the output value of the upstream Gaussian function ${\bar{y}}_{i}^{2}\left(N\right)$ is ${\bar{y}}_{i}^{3}\left(N\right)$ , meaning that the *j*-th node has complete and valid feature information. ${\bar{y}}_{i}^{2}\left(N\right)$ is fully input to the next layer. If ${\bar{a}}_{i}\left(N\right)$ is between ${\bar{T}}_{i}\left(N\right)$ and 1, it means that the *j*-th node has partially valid feature information and the input into the next layer should be the product of ${\bar{a}}_{i}\left(N\right)$ and ${\bar{y}}_{i}^{2}\left(N\right)$. If the value of ${\bar{a}}_{i}\left(N\right)$ is less than or equal to ${\bar{T}}_{i}\left(N\right)$, then the feature information of the *j-*th node is an invalid feature value, in which case ${\bar{y}}_{i}^{2}\left(N\right)$ will not be transmitted to the next layer. The output of the *T*-2 type Gaussian subordination function will be fed back to the next layer in whole or in part, depending on the evaluation of the feature information, and will not even be transmitted to the next layer [13].

The fourth layer is the fuzzy rule layer, where each node is connected to any of the three inputs corresponding to the third layer, respectively. Each input variable defines three levels, low, medium, and high, which constitute three fuzzy sets. Each node in this layer corresponds to a combination of different fuzzy sets, so there are 3^3^ = 27 nodes in this layer [14]. Each node corresponds to a fuzzy rule, and the input data on the node is fuzzy-operated according to equations (7) and (8).

(7) $$\begin{array}{c} {\bar{y}}_{k}^{4}\left(N\right)=\prod \omega_{j}^{4}{\bar{y}}_{j}^{3}\left(N\right)\text{,} \end{array}$$

(8) $$\begin{array}{c} {\underline{y}}_{k}^{4}\left(N\right)=\prod \omega_{j}^{4}{\underline{y}}_{j}^{3}\left(N\right)\text{.} \end{array}$$

The fifth layer is the defuzzification layer, in which two nodes perform the center-of-gravity method of defuzzification. The process in layer 5 can be described as the following expression:

(9) $$\begin{array}{c} y_{H}^{5}\left(N\right)=\frac{\sum_{k=1}^{n}\omega_{Hk}^{5}{\bar{y}}_{k}^{4}}{\sum_{k=1}^{n}{\bar{y}}_{k}^{4}}=W_{H}Y_{H}\text{,} \end{array}$$

(10) $$\begin{array}{c} y_{L}^{5}\left(N\right)=\frac{\sum_{k=1}^{n}\omega_{Lk}^{5}{\bar{y}}_{k}^{4}}{\sum_{k=1}^{n}{\bar{y}}_{k}^{4}}=W_{L}Y_{L}\text{.} \end{array}$$

The node defuzzification operation is shown in equations (9) and (10), where *y*_*L*_^5^(*N*) and *y*_*H*_^5^(*N*) are the node outputs of the fifth defuzzification layer. *W* is the center of mass of the set of type two.

The sixth layer is the output layer; the main purpose of equation (11) is to achieve linear integration between *y*_*H*_^5^ and *y*_*L*_^5^.

(11) $$\begin{array}{c} y_{o}^{6}=\eta y_{H}^{5}+\left(1-\eta \right)y_{L}^{5}=\eta W_{H}Y_{H}\left(\mu ,\bar{\sigma },\bar{\beta },\omega_{r}\right)+\left(1-\eta \right)W_{L}Y_{L}\left(\mu ,\underline{\sigma },\underline{\beta },\omega_{r}\right)\text{,} \end{array}$$

where *y*_*o*_^6^ denotes the output of T2RFS-FNN, i.e., the hysteresis threshold at the future moment.

### 3.2. Network Model Training

The structure of T2RFS-FNN can be seen as a multilayer feedforward network and, therefore, can be trained with errors by using the gradient descent method, just like BP networks. The RSRP, RSRQ, and throughput data received from the target base station are extracted as the training dataset based on the measurement reports reported by the high-speed train as it travels from the source base station to the target base station and are also used as the input to the FL system [15] to calculate the expected values. The same training dataset is used as input to the T2RFS-FNN, and for each set of training data input, the actual output value is calculated, and the error is calculated against the expected value. In the direction of error reduction, the weights are corrected layer by layer from the sixth layer of the network forward, and learning is continued to reduce the error until it is infinitely close to zero, then training is completed.

The error between the actual value and the expected value is calculated as shown in equation (12):

(12) $$\begin{array}{c} e=\frac{1}{2}{\left[y\left(t+1\right)-y_{d}\left(t+1\right)\right]}^{2}\text{,} \end{array}$$

where *y* (*t* + 1) and *y*_*d*_ (*t* + 1) denote the actual and expected values at the moment (*t* + 1), respectively. The weights at the moment (*t* + 1) are calculated from the weights at moment *t*.

## 4. Handover Algorithm Performance Indicators

In order to verify the performance of the algorithm in this paper, ping-pong handover and crosszone handover success rates are used as performance metrics.

### 4.1. Ping-Pong Handover

Ping-pong handover refers to the phenomenon of mobile terminals handover back and forth between the serving cell and the adjacent cell. When the train is switched to the target gNB base station, if the signal strength of the source base station and the signal strength of the target base station at this time satisfies equation (13) within the lag time, the ping-pong handover is triggered.

(13) $$\begin{array}{c} {PS}_{a}-{PS}_{b}\geq Hys\text{,} \end{array}$$

where PS_*a*_ and PS_*b*_ are the signal strengths of the source and target cells received by the train, respectively.

### 4.2. Handover Success Rate

If the train still satisfies the handover conditions after the trigger time delay TTT, the train executes the crosszone handover. In general, the success rate is defined as the probability that the train does not experience a communication interruption before triggering the handover and the probability that no communication interruption occurs even after the successful execution of the handover is completed since the following conditions are met [16]. Where a communication interruption is indicated as a communication interruption due to poor quality of the received signal [17]. Assuming that the signal-to-noise ratio threshold of the base station is *γ*, if the received signal quality is less than *γ* then a communication interruption occurs; the control-plane and user plane interruption probabilities are calculated as shown in equations (14) and (15):

(14) $$\begin{array}{c} P_{Cbreak}=P\left[SQ<\gamma \right]=Q\left(\frac{PS-I-\gamma_{tm}}{\sigma_{n}}\right)\text{,} \end{array}$$

(15) $$\begin{array}{c} P_{Ubreak}=P\left[SQ<\gamma \right]=Q\left(\frac{PS-I-\gamma_{ts}}{\sigma_{n}}\right)\text{.} \end{array}$$

In the abovementioned equation, SQ is the signal quality of the current service cell received by the train, *I* is the interference signal strength of the cochannel base station, and *σ*_*n*_ is the standard deviation of shadow fading, and then the success rate of the user-plane handover is defined as shown in equation (16):

(16) $$\begin{array}{c} P_{Usuccess}=\left[1-P_{break+}\right]∙P_{hando\;ver}∙\left[1-P_{break-}\right]\text{.} \end{array}$$

Since control-plane handover and user-plane handover are independent of each other, control-plane handover is only successful when both user-plane handover and control-plane handover are successful, so the success rate of control-plane handover is as shown in equation (17):

(17) $$\begin{array}{c} P_{Csuccess}=P_{Usuccess}∙P_{csuccess}\text{,} \end{array}$$

where *P*_*c*success_ is the handover success rate between macrobase stations in the control plane.

## 5. Experimental Results and Analysis

### 5.1. Parameter Configuration

This paper uses MATLAB software to build the proposed model and compare the performance of the algorithm. The main parameter configurations in the simulation experiment are shown in Table 1.

### 5.2. Predictive Performance Analysis

After the prediction parameters have been configured, the proposed prediction model is trained iteratively below. The RMSE is used to evaluate the predictive capability of the model.

**Figure 6:**
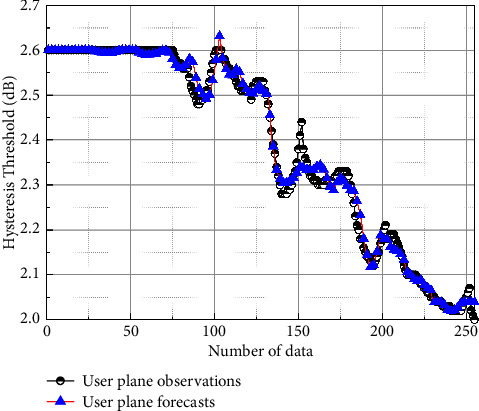
Comparison of predicted results for user-plane hysteresis thresholds.

**Figure 7:**
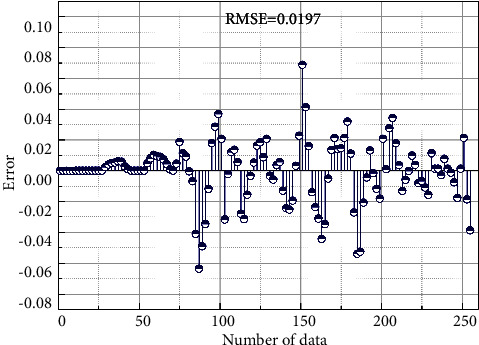
Prediction error of user-plane hysteresis threshold.

To test the prediction capability of the proposed dynamic prediction network for the transgressive handover hysteresis parameters in this paper, the optimized hysteresis threshold sequence of the fuzzy logic system was selected as the test data to verify the prediction effect, and the prediction results are shown in Figure 6. Figure 6 shows the prediction results of the threshold values of the trained network for the user plane compared with the actual observed values, and the blue curve in the figure shows the prediction results. The predicted values obtained by the method in this paper basically match the actual observed values, thus illustrating the effectiveness of the proposed model. In addition, Figure 7 shows the prediction error of the user plane hysteresis threshold, from which the mean squared error of the model in the prediction process of this paper is 0.0197.

**Figure 8:**
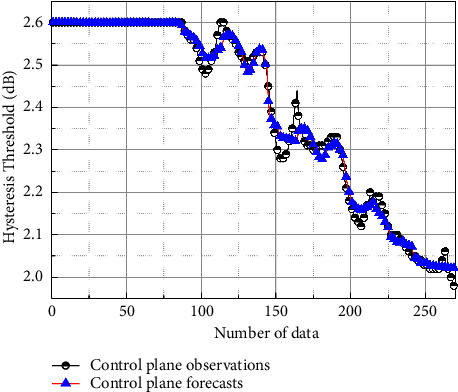
Comparison of predicted results for control-plane hysteresis thresholds.

**Figure 9:**
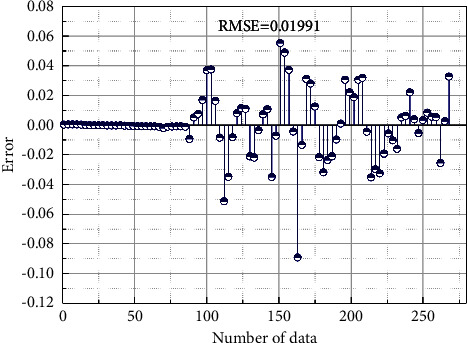
Prediction error of the control-plane hysteresis threshold.

Figure 8 shows the comparison between the predicted results of the control-plane threshold value and the actual observed values by the method in this paper; the blue curve in the figure is the predicted result. Figure 8 shows that the prediction results basically match the observed values, which illustrates the effectiveness of the method in this paper.  Figure 9 shows the prediction error of the control-plane hysteresis threshold predicted by the algorithm in this paper, and the mean square error value is 0.01991, indicating that the prediction effect of the algorithm on the hysteresis threshold value in this paper is good and can meet the requirement of high precision of the prediction of the hysteresis threshold [18].

### 5.3. Algorithm Simulation Analysis

**Figure 10:**
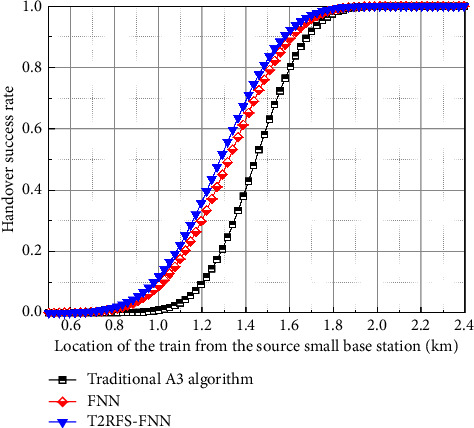
Comparison of the handover success rate of the user-plane handover algorithm.

In this paper, corresponding handover algorithms are designed for the control plane and user plane, respectively. The handover algorithm is selected from the traditional A3 algorithm, the FNN-based crosszone handover algorithm, and the algorithm in this paper for algorithm simulation analysis.  Figure 10 shows the comparison of the handover success rate of the user-plane handover algorithm; the success rate of the optimized crosszone handover algorithm is higher than that of the traditional A3 handover algorithm and the FNN crosszone handover algorithm because the algorithm of this paper considers the three factors of RSRP, RSRQ, and throughput. Degree function is to improve the processing capability of the model for nonlinear data while adding a feature selection layer to improve the prediction accuracy so that the threshold value of high-speed trains can be better adjusted according to the channel environment when handover at the user plane, stabilizing the handover performance of trains, and better solving the problem of low handover success rate due to the fixed handover hysteresis threshold value of the A3 algorithm; in addition, it can be seen from the figure that with the increase of *x*-axis, that is, the distance between the train and the source small base station is getting farther and farther, and the handover success rate is gradually increasing. This is because as the train runs, the train gradually moves away from the source small base station and approaches the target small base station, and the quality of service of the target small base station such as RSRP and RSRQ is obviously improved at this time; thus, the success rate of crosszone handover gradually increases. This conclusion is consistent with the findings of the study in [10], which further illustrates the effectiveness of the method in this paper.

**Figure 11:**
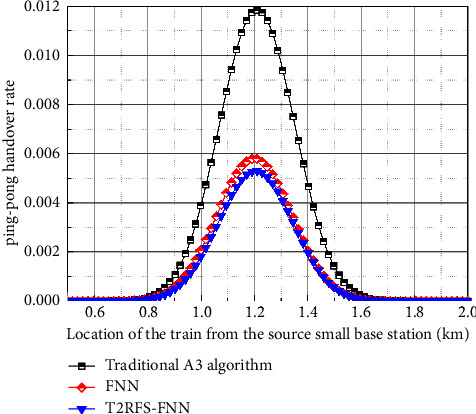
Comparison of the ping-pong handover rate of user-plane handover algorithm.

Figure 11 shows a comparison of the ping-pong handover rate of the user-plane handover algorithm where the ping-pong handover generally occurs near the middle region of the overlapping area of the overzone handover, and the ping-pong handover occurs when the train handover to the target base station and then cuts back to the source base station in reverse. If the ping-pong handover rate is larger, it means that the more unstable the performance of the crosszone handover algorithm is, the greater its adverse impact on the crosszone handover [19]. Compared with the FNN-based handover algorithm, the ability of this algorithm to express nonlinear data, such as the hysteresis threshold, is higher than that of the FNN-based handover algorithm, indicating that the handover performance of this proposed algorithm is optimal, while the traditional A3 algorithm has the highest ping-pong handover rate, indicating that it cannot continuously provide stable handover services for train crossing handover and is difficult to meet the handover requirements.

**Figure 12:**
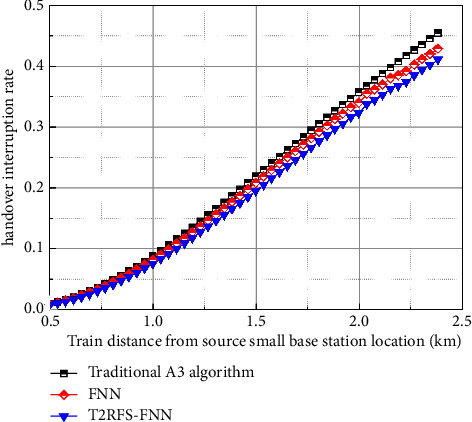
Comparison diagram of handover interruption rate of user-plane handover algorithm.

Figure 12 shows the comparison of the interruption rate of the user-plane handover algorithm, where the *x*-axis is the location of the train from the source small base station and the vertical coordinate is the probability of interruption of the handover, as shown in the figure, and the probability of interruption of the handover of the proposed algorithm in this paper is lower than that of the traditional A3 algorithm and the FNN-based handover algorithm; this is because the algorithm in this paper takes RSRP, RSRQ, and throughput factors into account during handover, so compared to the comparison algorithm can adjust the appropriate threshold for handover, effectively reducing the handover interruption probability caused by premature handover.

**Figure 13:**
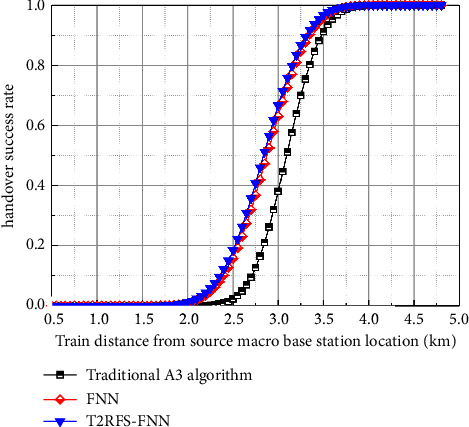
Comparison of the handover success rate of the control-plane handover algorithm.

Figure 13 shows a comparison of the success rate of the control-plane handover algorithm, where the control-plane and user-plane handover are relatively independent, i.e., the user-plane handover is completed before the control-plane handover, so the control-plane handover is performed with a relative lag compared to the user-plane handover. It can be seen from the graph that the handover success rate of the algorithm in this paper is higher than that of the comparison algorithm because compared with the FNN-based handover algorithm, the algorithm in this paper uses an interval type II affiliation function on the basis of FNN, and the values of the affiliation are mapped to intervals, which increases its randomness and improves the nonlinear expression capability of the algorithm, and at the same time, adds a feature selection layer to select data with valid features for input, which effectively reduces the prediction error of the model. The model prediction error is reduced, so the handover performance is optimal.

**Figure 14:**
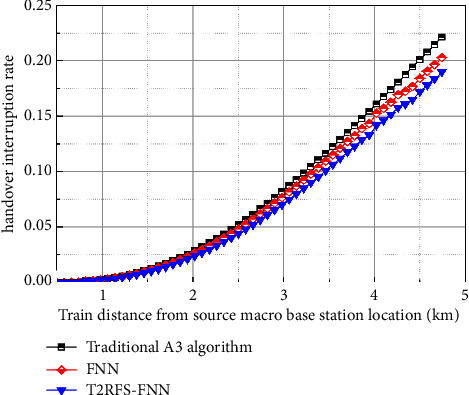
Comparison diagram of handover interruption rate of control-plane handover algorithm.

Figure 14 shows the comparison of the handover interruption probability of the control plane handover algorithm. As shown in Figure 14, the interruption probability of the control plane handover algorithm proposed in this paper is lower than that of the comparison algorithm because the processing capability of the control plane handover algorithm proposed in this paper for nonlinear data is higher than that of the comparison algorithm, and the optimization of the handover threshold is better, so the interruption probability of the algorithm in this paper is lower.

**Figure 15:**
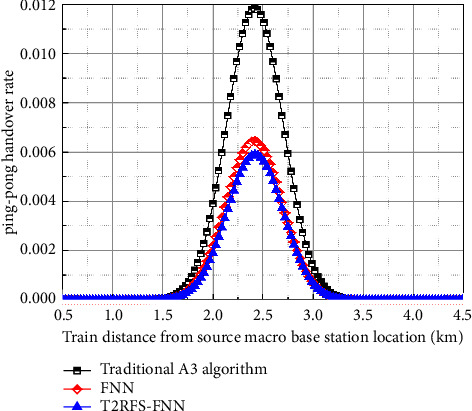
Comparison chart of ping-pong handover rate of control-plane handover algorithm.

Finally, the ping-pong handover rate of the control plane handover algorithm is analyzed, as shown in Figure 15. In the central region of the handover overlap area where ping-pong handover is frequent, the ping-pong handover rate of this algorithm is lower than that of the comparison algorithm, indicating that the handover performance of this algorithm is stable and can provide stable handover services for train crossing handover.

## 6. Conclusions

1. In response to the problems of fixed hysteresis thresholds and single considerations in traditional handover algorithms, this paper proposes a crosszone handover optimization algorithm based on interval type 2 feature selection recurrent fuzzy neural networks. A feedforward neural network structure is used to implement FL inference, and an interval type II Gaussian subordination function is used to improve the nonlinear expression capability of the model, while a feature selection layer is added to determine the output of the subordination function and uncertainty inference is used to carry out fuzzy inference to complete the optimization of the hysteresis threshold.
2. The inputs to the network model are adjusted to account for the functional differences between the control plane and user plane, and the hysteresis threshold is optimised for the control-plane handover algorithm by considering RSRP and RSRQ, and for the user-plane handover algorithm by considering RSRP, RSRQ, and throughput.
3. This method outperforms the traditional A3 handover algorithm in terms of handover success rate, handover interruption rate, and ping-pong handover rate, solving the problem of fixed handover parameters and single consideration of the traditional method, and effectively improving the handover performance. The research results can provide some theoretical reference basis for high-speed railway train speedup and 5G-R evolution.

## Figures and Tables

**Table 1:** Parameter settings of the experimental test.

Name	Specific parameters
Macrobase station carrier frequency	2 GHz
Small base station carrier frequency	5 GHz
Macrobase station transmitting power	43 dBm
Small base station transmitting power	33 dBm
Macrobase station signal-to-noise ratio threshold	18 dB
Small base station signal-to-noise ratio threshold	15 dB
Shadow decay bias	4 dB
Fixed hysteresis threshold	3 dB
Macrobase station antenna height	30 m
Small base station antenna height	5 m
Macrobase station path loss model	COST231-Hata
Small base station path loss model	WINNER-IID2a

## Data Availability

The data used to support the findings of this study are available from the corresponding author upon request.

## References

[1] Cai X., Wu C., Sheng J., Wang Y., Ai B. (2022). Spectrum situation awareness based on time-series depth networks for LTE-R communication system. *IEEE Transactions on Intelligent Transportation Systems*.

[2] Pang M. M. (2021). Research on core network architecture of railway 5G-R system. *China Railway*.

[3] Mollel M. S., Abubakar A. I., Ozturk M. (2021). A survey of machine learning applications to handover management in 5G and beyond. *IEEE Access*.

[4] Liang G., Yu H., Guo X., Qin Y. (2019). Joint access selection and bandwidth allocation algorithm supporting user requirements and preferences in heterogeneous wireless networks. *IEEE Access*.

[5] Yan L., Fang X., Fang Y. (2017). A novel network architecture for C/U-plane staggered handover in 5G decoupled heterogeneous railway wireless systems. *IEEE Transactions on Intelligent Transportation Systems*.

[6] Cheng J., Zhang Y., Wang F. Seamless 5G handover scheme based on C/U plane separation and beamforming in HSR environment.

[7] Zeng X. B. (2020). *Research and optimization of 5G system handover scheme in high-speed rail scenario*.

[8] Ma R. (2021). *Handover Optimization of 5G C/U Split Heterogeneous Network in High-Speed Railway*.

[9] Li L. H. (2018). Research on handover technology of LTE system in high-speed railway environment.

[10] Liu Y. Y. (2018). Research on handover technology in LTE-R for high-speed railway environment.

[11] Cui E. J. (2021). *Research on Handover Optimization in High-Speed Railway Wireless Communication System*.

[12] Li C. R., Xie J. L., Gao W. J. (2021). Heterogeneous network access technologies based on 5G-R services for high-speed railways. *ZTE Technology Journal*.

[13] Hou S. X., Chu Y., Fei J. (2021). Adaptive type-2 fuzzy neural network inherited terminal sliding mode control for power quality improvement. *IEEE Transactions on Industrial Informatics*.

[14] Ma B., Wang S., Chen H. B. (2021). Vertical handover algorithm based on interval type-2 fuzzy neural network. *Acta Electronica Sinica*.

[15] Chen Y., Niu K. Y. (2022). Adaptive handover optimization algorithm for LTE-R based on fuzzy logic. *Journal of Railway Science and Engineering*.

[16] Batlle Franch P., Josep Eritja Olivella A., Maria Garcia Alarcia R., Dini P. User mobility inference and clustering through LTE PDCCH data analysis.

[17] Akhpashev R. V., Andreev A. V. COST 231 Hata adaptation model for urban conditions in LTE networks.

[18] Kurri V., Raja V., Prakasam P. (2021). Cellular traffic prediction on blockchain-based mobile networks using LSTM model in 4G LTE network. *Peer-to-Peer Networking and Applications*.

[19] Tong H. N., Wang T., Zhu Y., Liu X., Wang S., Yin C. (2021). Mobility-aware seamless handover with MPTCP in software-defined HetNets. *IEEE Transactions on Network and Service Management*.

